# Polio-Myilitis

**Published:** 1893-10

**Authors:** J. T. Eskridge

**Affiliations:** Denver, Colorado, Professor of Nervous and Mental Diseases in the Medical Department of the University of Colorado, Alienist and Neurologist to the Arapahoe Co., the St. Luke’s and the Deaconess’ Home Hospitals


For Texas Medical Journal.

### POLIO-MYELITIS.

### A Clinical Lecture Delivered at the Arapahoe County Hospital, Colorado.

BY J. T. ESKRIDGE, M. D., DENVER, COLORADO,
Professor of Nervous and Mental Diseases in the Medical Department of the University of Colorado, Alienist and Neurologist to the Arapahoe Co., the St. Luke's and the Deaconess' Home Hospitals.

GENTLEMEN:—We will study to-day the case of J. H., a large, well developed, muscular man, aged 46, laborer, Fenian, family history unimportant, came to America nine years ago. He has lived two years in California, three years in Montana and four years in Colorado. He has never had any illness, with the exception of syphilis fifteen years ago, until this summer, when he had mountain fever, at Ouray, Colorado. He states that he was sick six weeks with it. After that he returned to his work. Four weeks ago yesterday he felt his right wrist suddenly become cold and stiff. He rubbed it with cold water; it got better. He went on working, had no pain, but felt that something was wrong with the right arm. The next morning when he got up it was stiff. He worked it up and down, it got a little better, and he went to work. He had a little pain in the upper part of the right arm. At night the pain got worse and it felt as if needles were being put in it. He did not sleep. The next morning his right hand and arm were weak. He worked, but had to rest his arm every few minutes. He had pain in the muscles, but it was not very bad. The next day he tried to work again, but had no power in his right hand to hold anything, so that he had to lay off, and has not worked since.

EXAMINATION:—No ataxia in legs. The gait is perfect. He walks normally with his eyes closed. Putting him in all positions for testing ataxia, shows that the ability to co-ordinate is perfect. Knee-jerks, R. increased, and but little influenced by re-inforcement; L. greater than right, but little increased by re-inforcement. Ankle clonus; R. absent; L. There is a light tendency toward ankle clonus in the left, but not decided. There is only a little quivering of the muscles when the attempt is made to elicit ankle-clonus on this side. Plantar reflexes; R.

very slight, but trying to elicit it causes some nervous movements of the leg muscles and even those of the opposite leg. The reflex causes dorsal flexion of foot. L. Instead of plantar flexion we have dorsal flexion with contraction of all the muscles of the left leg. Cremaster reflexes are both present. Abdominal reflexes, both lower and upper, are present. There is an hyperæsthetic condition over the abdomen. We find all the muscles of the legs strong and well developed. Pupils are slightly large; they react to light and accommodation. He is unable to move the indicator of the dynamometer with the right hand; the left hand registers 130. I find all the muscles of the left arm strong. The right hand can be closed and opened, but the movements are exceedingly weak. The extensors of the hand are weaker than the flexors. The flexors and extensors of the wrist are weak, the former more so than the latter. The extensors and flexors of the elbow are weaker than those of the wrist. Supination and pronation are performed very well. The triceps and biceps are almost completely paralyzed, the triceps being weaker than the biceps. There is absolute paralysis of the right deltoid muscle, and of the upper or clavicular fibres of the pectoralis major. These muscles are the only ones that are absolutely paralyzed. Flexors and extensors of the wrist are stronger than those of the elbow, and flexors and extensors of the fingers are stronger than those of the wrist, so that the further we go toward the distal portion of the limb the less complete we find the paralysis.

In passing, I wish to call your attention to the condition of the great pectoral muscle in this case. You all know that this muscle is divided into two parts, the inferior or sternal portion, and the superior or clavicular portion. The inferior portion acts independently of the superior in depressing the arm; and the superior acts independently of the inferior in elevating the arm above the horizontal position, whilst both divisions of the muscle act in association with each other in carrying the arm across the chest. It has been found by Beevor that one portion of this muscle may be paralyzed for its separate function in depressing or elevating the arm, as the case may be, yet this paralyzed portion may contract quite vigorously when acting in association with the other portion in carrying the arm across the chest.

In the patient before us, the inferior portion of the right great pectoral muscle contracts quite well in forcibly depressing the arm against any obstacle that may be thrown in its way, but no fibres of the superior portion of the muscle contract when he make fruitless efforts to raise the arm above the horizontal position. The deltoid is paralyzed, and I have to raise and maintain the arm in the horizontal position, so as to give the superior portion of the great pectoral muscle the most favorable condition for contracting. If I now ask him to bring the right arm, extended in the horizontal position, across the front of the chest, and at the same time offer resistance to this movement of his arm, both the lower and upper portions of the great pectoral muscle contract. We find then, that the superior, or clavicular, portion of the right great pectoral muscle is paralyzed for movements performed independently of the inferior portion, but not so for associated movement in bringing the arm across the front of the chest.

To the significance of dissociated paralysis I will direct your attention when we come to consider the diagnosis of this man's trouble. The middle portion of the right trapezius muscle is weak if not completely paralyzed.

MEASUREMENTS:—Hands, R. 8½; L. 7⅞. Forearm, R. 10; L. 9¾. Upper arm, R. 10¼; L. $10\frac{3}{16}$. Over the biceps the left arm is smaller than the right. Over the deltoid, R. 10⅜; L. 10⅞. Touch, pain, temperature, muscular, pressure and posture senses are normal. There has been no pains in the joints, but some pains have been experienced in the muscles of the right arm. The pain is limited to the point I touch, and does not shoot down the limb. The nerves of the arm are not sensitive to pressure. The muscles of the right arm respond almost as readily to the faradic current as do those of the left. The right deltoid, biceps, triceps and sternal portion of the great pectoral muscles show some comparative loss of irritability to the faradic, but this is so slight as to be scarcely noticeable.

Let us consider what lesions may give rise to symptoms somewhat similar to those presented by this patient: The first thing that suggests itself when we have paralysis in one arm is neurosis, and in a laboring man, pressure neuritis. Laborers are very prone to be injured in the nerves, especially of the arms.

Pressure neuritis of the arm most commonly is caused by persons lying with the head on the arm, especially while the latter is resting on some hard substance, by the use of crutches that fit in the axilla, or by any means by which undue and prolonged pressure is exerted over a nerve. The symptoms are both motor and sensory. The paralysis may be partial or complete, and is worse, as a rule, at the distal portion of the extremity. In ordinary pressure neuritis of the arm, caused by external agents, applied at the axilla, or below, the deltoid muscle is not involved. If the trouble is more than transient, the nerves of the arm become sensitive to pressure, and are the seat of spontaneous pain. The affected parts have a numb feeling, and not unfrequently swell. The absence of all sensory symptoms in the course of the nerves, the involvement of the deltoid muscle, and the presence of the greatest motor symptoms at the proximal portion of the extremity enable us to exclude pressure neuritis.

The absence of all sensory symptoms, in the course of the nerves, enable us to exclude neuritis of the entire brachial plexus, or of part of it, from a growth pressing on any of the nerves that compose it. We must remember that soon after a pressure neuritis has set in, and in some cases, for weeks or months afterward, the nerves below the seat of pressure are not sensitive to pressure, but they are, nevertheless, the seat of sharp, shooting pains, and numb sensations, and sometimes a condition of hyperæsthesia may be present throughout the entire limb, below the point where pressure is exerted, and this takes place before absolute paralysis.

Is the trouble from which this man is suffering cervical pachymeningitis?

The symptoms of this affection are almost always bilateral, and are sensory, motor and trophic in their nature. Pain is a prominent symptom, and it extends to the head as well as to the arms. The disease is gradual in its onset, and pain and trophic disturbances are usually pronounced before paralysis is obtrusive. In the case before us, the only well-defined symptom is paralysis, affecting the upper more than the lower portion of the right arm. There have been no bilateral symptoms. It does not seem difficult to be able to exclude cervical pachymeningitis.

Would a hemorrhage into the cord, or its membranes, account for the symptoms?

In hemorrhage into the substance of the cord, the symptoms are ushered in suddenly, and consist of paralysis and loss of sensation. In hemorrhage into the membranes, the symptoms are also sudden in their invasion, and consist of great pain in the muscles of the back and down the limbs, whose nerve distribution is affected; paralysis is not absolute, and there is either anesthesia or hyperæsthesia, or both. The symptoms in the case before us were three or four days in developing, and paralysis has been complete in certain muscles, with no hyperæsthesia or anesthesia, and with but little pain. Hemorrhage into the cord, or its membranes, can be excluded.

Would transverse myelitis, affecting the cervial portion of the cord, account for the symptoms?

In such a condition, besides having respiration affected, sensory disturbance would be well marked before paralysis became as complete as we find it in the case before us. The symptoms would be bilateral. We can then exclude transverse myelitis.

There seem to be but two lesions left that might give rise to local paralysis, somewhat resembling the symptoms presented by our patient, viz.: A localized cerebral, or spinal (polio-myelitis anterior acuta) affection. A cortical or sub-cortical lesion in the arm center, on the left side of the brain, would give rise to paralysis with little or no anæsthesia. In brain lesions the distal paralysis is usually greater than the proximal, just the reverse of what we have there. If the paralysis in the patient before us were of brain origin, its gradual onset of two or three days would exclude hemorrhage. If the cerebral lesion were a tumor, or abscess, we would likely have headache and other cerebral symptoms. Further, a gross lesion, such as tumor, or abscess in the arm center of the left side of the brain, is likely to indirectly affect the face center, and, to some extent, modify speech. Did we need further evidence in excluding brain lesion in this case? We have it in the wasting of the right deltoid muscles, its lessened response to the faradic current, and the presence of dissociated paralysis, to the significance of which I wish now to direct your attention.

Dr. Charles E. Beevor, in Journal Brain for 1891, p. 51, has recorded some interesting observations on the action of muscles. After considering the action of muscles at some length, he puts this query: "What is the diagnostic value of paralysis in a muscle for one movement and not for another?"

This question follows almost as a corollary to the preceding one. It will be obvious, I think, that this condition of a muscle, or group of muscles, can only be brought about by a central lesion, as it would be difficult to imagine that a disease of the muscle fibres, such as occurs in pseudo-hypertrophic paralysis, and other myopathics, or neuritis of the peripheral nerve, between the brachial plexus and the muscle, could affect one class of movements of a muscle more than another, we should expect them to be diminished or paralyzed equally. With regard to central diseases, I am not aware, at present, of any case of disease of the cortex cerebri, or pyramidal tract, in which the lesion was so localized as to cause paralysis of the muscles elevating the shoulder and not those depressing it, but it is in disease of the spinal cord that the condition is best shown. Cases of poliomyelitis and amyotrophic lateral sclerosis, have already been mentioned, and I have also seen cases of infantile paralysis and progressive muscular atrophy, showing the same condition. Though I have not had the opportunity of examining a case of injury to the cervial roots, before they join the plexus, I think we are justified in assuming, from the resemblance of the grouping of the paralyzed muscles with those due to central spinal disease, that the same condition would be found.

"The position of the trophic centres in the brachial enlargement of the cord for the clavicular fibres of the pectoralis major, is an interesting question, in connection with the second of my cases related above—i. e., that of the boy; for, although the muscle was paralyzed for movements associated with the deltoid, its trophic centre was not sufficiently affected to give any reaction of degeneration with either form of electricity. Whether the trophic centre for the clavicular fibres is in quite the same part of the cord as for the sternal flbres, seems doubtful from a case of infantile paralysis, which I have previously observed and recorded, where the sternal fibres were completely absent, and yet the clavicular fibres were well developed and reacted normally to both currents.

"From the above consideration I think we have another symptom to enable us to dignose, on the one hand, between central disease affecting the spinal cord, and on the other, lesions affecting the peripheral nerves and the various myopathies, while the condition of a muscle, being paralyzed for one movement and not for another, is in favor of the grouping of the muscles in the brachial and lumbar enlargements, being rather of a physiological than a purely anatomical nature."

In referring to these facts, he states in his fifth conclusion: "And this condition would point to the lesion being in the spinal cord, or its roots, and not in the peripheral nerves or the muscles, primarily."

I have read at some length from Dr. Beevor's excellent paper, because of the importance of hls conclusions if his observations are correct. The results of his investigations in the direction of dissociated paralysis of certain fibres of muscles in relation to other fibres of the same muscles, may be summed up in one sentence,—when certain fibres of a muscle have two actions, one to contract in association with other fibres of the same muscle, and the other to contract independently of these fibres, and the muscular fibres should be paralyzed for one of these movements, but not for the other, the lesion is in the spinal cord and not in the brain or nerves.

Having excluded all other lesions of the nervous system whose symptoms would likely be confounded with those presented by our patient, we have left polio-myelitis anterior acuta affecting one anterior horn of the cervical region of the cord. The significant sympton of this disease is paralysis involving one or more limbs, sometimes sudden, but more commonly rapid in its onset. Not infrequently the paralysis is two to four days or more in reaching its height. There are, as a rule, no objective sensory disturbance. The subjective sensory symptoms of anterior polio-myelitis are, tingling, numb sensations, and sometimes, pains in the muscles, but, according to Gowers, the joints are unaffected.

We must bear in mind that we sometimes have multiple neuritis, complicating polio-myelitis. The deep reflexes in uncomplicated cases are abolished in the paralyzed muscles, but uninvolved either above or below the cord lesion. How then are we to account for increased knee-jerks which our patient presents?

In some cases the pyramidal fibres nearest the anterior horns are irritated, when the deep reflexes below the cord lesion will show an increased irritability. It is probable that the pyramidal tracts have been irritated in our patient, thus accounting for the exaggerated knee-jerks which we find in his case.

Another point in the diagnosis of polio-myelitis worthy of attention is, that among the arm muscles the deltoids usually suffer most, and the supinators sometimes escape, a condition that we find in the patient whose case we have studied to-day.

What have we to guide us in the prognosis of this man's disease? The muscular wasting and the faradic irritability of the nerves supplying the affected muscles are our best guides in regard to prognosis, and of these two, the degree of the faradic irritability is the most important. Lessened faradic irritability in severe cases may be manifest by the fourth or fifth day, and may be lost by the end of first week. In all such cases the wasting will be considerable, and the recovery incomplete. In some cases the faradic irritability does not begin to lessen before the tenth day. In such cases, if it is not long progressive after it becomes apparent, there will be but little wasting, and recovery will take place, although probably not complete, in a few months. In some cases there is little or no loss of irritability to the faradic current. In these cases recovery will take place, and be more or less complete, in a few weeks or months.

In the patient before us there is only slight loss of faradic irritability, and this is limited to the nerve (circumflex) supplying the deltoid muscle. It is now four weeks since the affection began, so that we can with considerable assurance predict that our patient will recover so as to be able to return to work in four or five weeks.

Wasting usually takes place by the end of the second week, but in the man before us at the end of the fourth week, there is only slight wasting, and this is limited to the deltoid muscle. It is probable then, judging from the muscular nutrition and the preserved faradic irritability of the nerves that wasting will progress no further.

What can be done in the way of treatment?

In all cases of polio-myelitis, no matter how limited or slight the lesion, absolute rest in bed should be insisted upon from the first inception of the trouble to the end of the inflammatory stage. It is just possble that if this patient had stopped work and sought medical advice when the symptoms of the disease first made their appearance, the lesion might have been less severe than it is to-day. As soon as the disease can be localized to any particular portion of the cord, leeches applied to the spine over this region seem to be indicated. At the same time counter-irritation, either by means of alternate application of cold and hot substances, by the application of mustard plasters or by blisters, aid in relieving the spinal cord engorgement by drawing the blood to the surface. If we bear in mind that the blood from the tissues covering the spines and from the bones themselves, empties into the spinal canal to pass through the spinal plexus of veins we shall appreciate why counter-irritation relieves the amount of blood in the spinal canal, including the spinal cord, as the blood from the latter finds its way into the spinal venous plexus. At the outset of the disease the bowels should be thoroughly evacuated by five or ten grains of calomel, and later, until the acute stage is over, a gentle impression of calomel in one-eighth of grain doses should be kept up. Ergot has been recommended in this disease, and I advise you to try it in half drachm doses every three or four hours. The patient should be encouraged to lie on the side or abdomen as much as possible, so as to lessen by gravity the amount of blood in the spinal canal.

After the acute stage of the disease has passed, as we find it in the patient before us, the treatment should be different from that just outlined. In this tonics, good food, massage and electricity are serviceable. The nerve tonic which seems to be most indicated immediately following the acute stage is arsenious acid in $\frac{1}{100}$ to $\frac{1}{60}$ grain doses thrice daily, given after meals. Later, quinine, strychnine and cod liver oil are excellent. The patient should be kept well nourished by proper food.

Massage may be resorted to in mild cases by the end of the second week, in severer cases it is not well to employ it until all inflammation has subsided. At first it should be given gently, and the affected muscles only should be manipulated, and these only for a short time, but later, the entire body should be massed and the manipulation may be thorough, and prolonged for an hour.

In regard to electricity, this should not be applied before the stage of inflammation has subsided. At first the affected muscles should not be made to contract more than fifty or sixty times at each sitting, which should not be oftener than every second day. Later, the application of electricity may be made daily, and the seance prolonged for an hour. In regard to the choice of the current to be used, between the galvanic and faradic, the latter probably is as efficient as the former when a response of the muscles is elicited by a current of medium strength. When the faradic current fails to cause contraction of the paralyzed muscles, its application is worthless.

N. B. The patient soon began to show improvement. By the end of the second week the fore-arm muscles showed considerable gain in strength, and by the end of the third week the deltoid was able to bring the arm to the horizontal position. He was discharged practically cured in less than two months from the time he entered the hospital.

---

**Frank R. Stockton** has written the history of "How I Wrote 'The Lady, or the Tiger,'" for the next issue of *The Ladies' Home Journal*, and tells what came of the writing of the famous story, and the condition of his own mind, at the present time, of the correct solution of the problem, whether the lady or the tiger came out of the open door.

